# Predicting the Posture of High-Rise Building Machines Based on Multivariate Time Series Neural Network Models

**DOI:** 10.3390/s24051495

**Published:** 2024-02-25

**Authors:** Xi Pan, Junguang Huang, Yiming Zhang, Zibo Zuo, Longlong Zhang

**Affiliations:** 1 General Engineering Institute of Shanghai Construction Group, Shanghai Construction Group Co., Ltd., Shanghai 200080, China; panxi@scg.cn (X.P.); zuozb@scg.cn (Z.Z.); zhangll@scg.cn (L.Z.); 2 School of Civil and Transportation Engineering, Hebei University of Technology, Xiping Road 5340, Tianjin 300401, China; yiming.zhang@hebut.edu.cn

**Keywords:** high-rise building machine (HBM), steel platform (SP), neural networks (NNs), multivariate time series (MTS), posture prediction

## Abstract

High-rise building machines (HBMs) play a critical role in the successful construction of super-high skyscrapers, providing essential support and ensuring safety. The HBM’s climbing system relies on a jacking mechanism consisting of several independent jacking cylinders. A reliable control system is imperative to maintain the smooth posture of the construction steel platform (SP) under the action of the jacking mechanism. Long Short-Term Memory (LSTM), Gated Recurrent Unit (GRU), and Temporal Convolutional Network (TCN) are three multivariate time series (MTS) neural network models that are used in this study to predict the posture of HBMs. The models take pressure and stroke measurements from the jacking cylinders as inputs, and their outputs determine the levelness of the SP and the posture of the HBM at various climbing stages. The development and training of these neural networks are based on historical on-site data, with the predictions subjected to thorough comparative analysis. The proposed LSTM and GRU prediction models have similar performances in the prediction process of HBM posture, with medians R^2^ of 0.903 and 0.871, respectively. However, the median MAE of the GRU prediction model is more petite at 0.4, which exhibits stronger robustness. Additionally, sensitivity analysis showed that the change in the levelness of the position of the SP portion of the HBM exhibited high sensitivity to the stroke and pressure of the jacking cylinder, which clarified the position of the cylinder for adjusting the posture of the HBM. The results show that the MTS neural network-based prediction model can change the HBM posture and improve work stability by adjusting the jacking cylinder pressure value of the HBM.

## 1. Introduction

**Figure 1:**
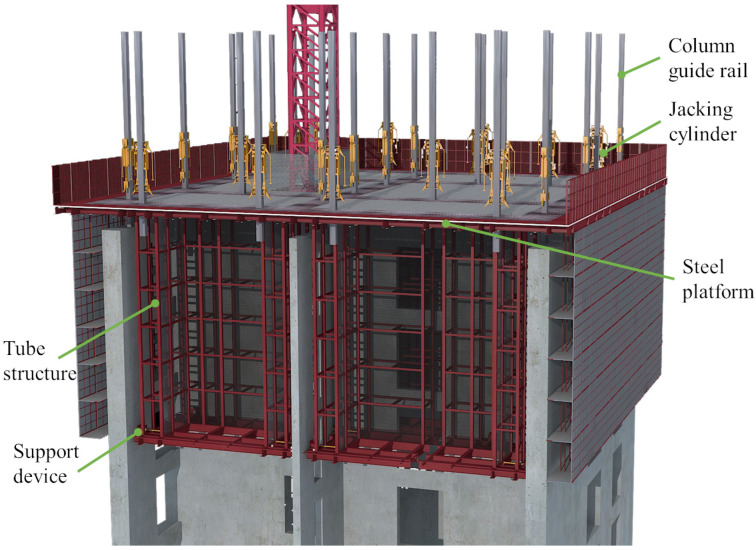
Schematic diagram of the HBM.

High-rise building machines (HBMs) are innovative self-elevating steel-structure-building machines designed for constructing super high-rise buildings [1,2,3,4]. They offer several advantages: high automation, enhanced safety, simplified construction organization, assured structural quality, and standardized processes [5,6,7]. These features contribute to accelerated construction progress. Figure 1 illustrates the three-dimensional structural details of the HBM [8,9,10]. The levelness of the steel platform (SP) represents the HBM’s posture changes [11,12,13]. During the climbing process of the HBM, the telescopic support device retracts from the structurally reinforced concrete shear wall, while the climbing system supports the SP to ascend along the column guide rail. Once the SP reaches the desired position, the telescopic support device is extended into the reserved hole of the structurally reinforced concrete shear wall. However, due to uneven stacking loads on the SP, the levelness of the SP may fluctuate. If excessive levelness deviation is not promptly corrected, the HBM may undergo severe shape distortion, leading to collisions with obstacles and halting the climbing [14]. In the early stages of HBM usage, workers employed the hanging-line-pendant method and plumbing instruments to measure the HBM’s verticality. They used jacks to lift parts with low levelness, applying corrective forces to gradually restore the SP’s levelness while continuing to climb [15,16]. Subsequently, climbing control operators monitored the HBM’s posture information directly via signal and image displays on the center console. Although this approach improved efficiency and reduced measurement errors, it incurred significant costs. The detection equipment enhanced production efficiency, minimized operational errors, ensured detection repeatability, and generated extensive monitoring data [17,18,19,20]. However, these measures still failed to prevent levelness deviations during the climbing process, addressing the complexity of repairing such deviations and mitigating subjective errors in worker operations. Zuo et al. conducted a study on the determination of concrete strength during the construction of HBMs by proposing a remote real-time monitoring system [10]. Pan et al. collected the vibration frequency of HBMs and utilized a machine learning method to categorize and assess the four primary working states of this machinery [9]. They also proposed a method for monitoring and issuing warnings during the climbing process of an HBM [8]. However, these methods are primarily focused on monitoring and providing early warnings for the safe operation of HBMs. There needs to be more in-depth research on predicting the future working state of this machinery and dealing with potential dangers.

We have discovered that a change in posture of a HBM exhibits a multivariate time series (MTS)-related process. To effectively capture the intricate patterns and dependencies in time series data, neural networks (NNs) are a valuable tool [21,22,23,24,25,26]. NNs possess linear and nonlinear fitting capabilities, making them adept at handling such data. Notably, NN technology has shown promising results in prediction tasks [27,28,29,30]. It is important to note, though, that these neural networks don’t do as well when they have to deal with tasks that use MTS class information and simulations that have strong back-and-forth correlations. Several articles from 2015 and 2020 have talked about recurrent neural networks (RNNs) [31,32,33,34,35]. These are a type of time series neural network that have memory cells and feedback connections that make it easier for information to flow within the model and handle strong back-and-forth dependencies well. Using a recursive structure, RNNs retain contextual information from past observations and apply it to current or future predictions. However, RNNs encounter challenges when dealing with long time series, leading to gradient vanishing or exploding issues. Consequently, traditional RNNs excel at solving short-term dependencies. Advanced architectures such as the Long Short-Term Memory (LSTM) [36,37,38] and the Gated Recurrent Unit (GRU) [39,40,41,42] have been developed to address this. LSTM employs memory cells and gating mechanisms to filter out the noise and selectively capture long-term dependencies more efficiently. On the other hand, GRU makes the LSTM structure simpler by only using hidden states to send information. This cuts down on parameters and computational complexity while keeping the performance the same in some situations. Not only are recurrent neural networks used to process time series data, but so are Temporal Convolutional Neural Networks (TCNs) [43,44,45,46]. TCNs modify the conventional approach of using two-dimensional convolutional kernels for image processing by employing one-dimensional convolutional kernels to extract local patterns and features. TCNs possess a large enough sensory field to find patterns and regularities across a range of time scales by stacking multiple convolutional layers with pooling operations.

Previous studies have demonstrated the remarkable capabilities of neural networks in forecasting time series data. This study is organized as follows: Section 2 provides a detailed explanation of the characterization of the HBM through sensor-based monitoring during its climb. It also discusses the preprocessing techniques employed to handle this data effectively. Moving on to Section 3, an in-depth exploration of three multivariate time series-prediction models, namely the LSTM, GRU, and TCN models, is presented. The technical approach adopted to predict the HBM’s posture in this study is also discussed. Section 4 delves into an insightful comparison between the prediction results of these three models and the actual values. It also includes a meticulous analysis of the potential sources of errors generated by the models. Finally, Section 5 and Section 6 present the discussion and conclusions drawn from the research. It is shown that the suggested smart-prediction system is better than the old ways of manipulating HBMs and reduces the hysteresis of repairing the HBM’s posture.

## 2. The Posture of the HBM

### 2.1. Data Sources

**Figure 2:**
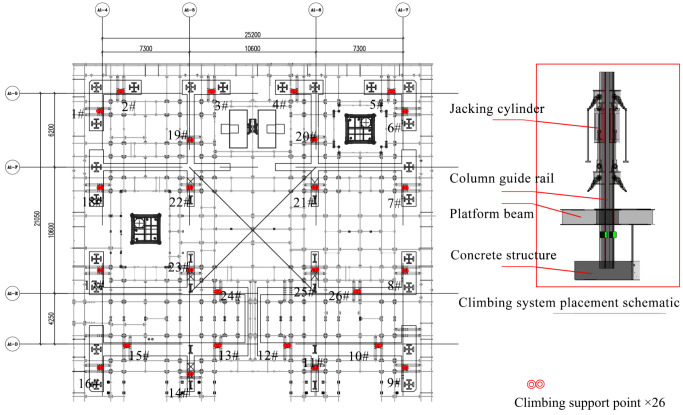
Schematic layout of jacking cylinders.

**Figure 3:**
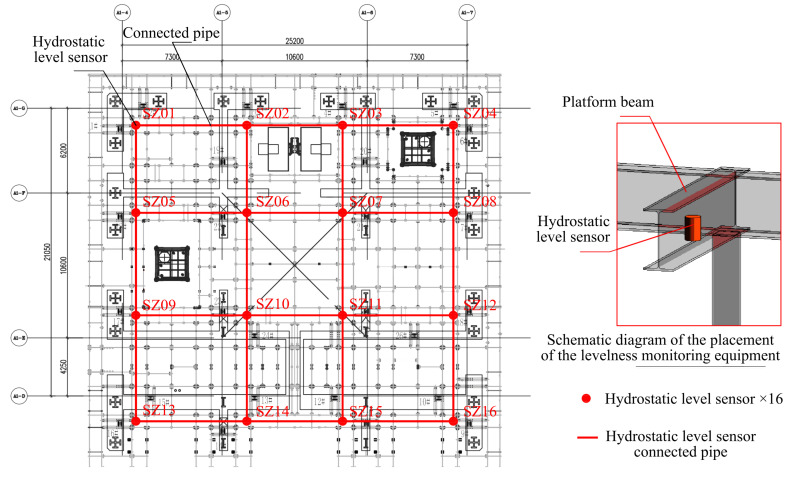
Schematic layout of levelness-monitoring equipment.

In this work, we observed and analyzed the HBM climbing process at the West Tower of the Shenzhen Xinghe Yabao Building project (356m) in China. With an average climbing frequency of once every five days, we embarked on an exploration utilizing a dataset comprising 30 sets (days) of monitoring data. Each dataset, sampled at a rate of one sample per second, encompassed 168,048 monitoring data points. These data points comprised 68 sample characterizations, forming a foundation for our investigation. As showcased in Figure 2, our monitoring efforts focused on observing and recording the jacking stroke and pressure of 26 climbing jacking cylinders throughout the climbing process. We also monitored the levelness of 16 crucial monitoring points on the steel platform beam, as shown in Figure 3.

### 2.2. The Pre-Processing of Monitoring Data

**Figure 4:**
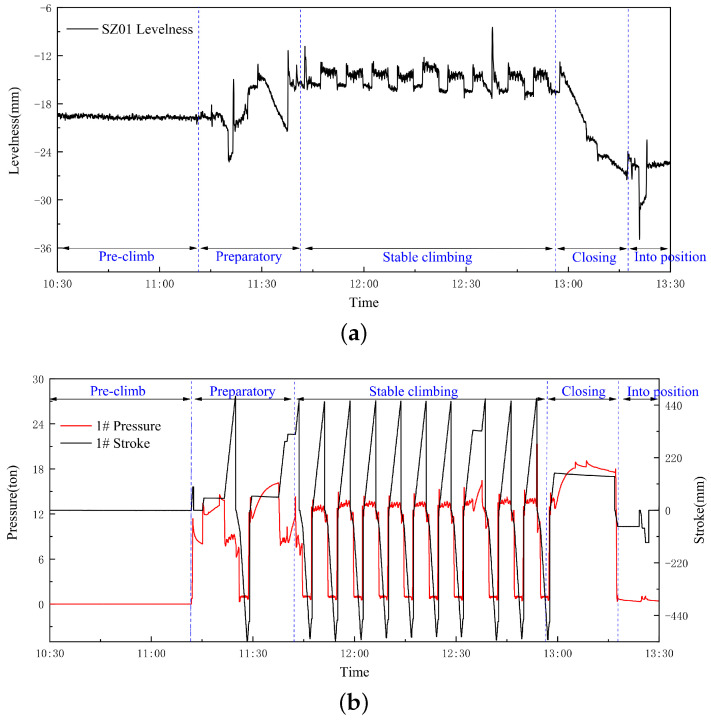
One set of measured time-range data during one climb: (**a**) levelness-sensor monitoring data, (**b**) jacking cylinder-monitoring instruments monitoring data.

Figure 4 depicts the once-climbing process of the HBM, presenting a narrative of the levelness of the SP, jacking cylinder jacking stroke, and jacking-cylinder pressure over time. This intricate climbing process can be divided into five distinct phases: the pre-climb stage, the preparation stage, the stable climbing stage, the closing of the climbing stage, and the climbing-into-position stage. Remarkably, the pre-climbing and climbing-into-position stages exhibited minimal changes in the posture of the HBM. On the other hand, the preparation stage and the closing of the climbing stage are influenced by equipment-operation errors arising from installing the jacking cylinders, such as support jacking cylinder position errors and jacking cylinder tilt. And, we want, as the climb progresses into the stabilized climbing stage, a gradual elimination of the levelness deviation of the SP to become evident. In addition, the initial levelness deviation observed in the stable climbing stage is not attributed to the climbing process itself. Consequently, our focus narrows to studying and analyzing the data solely from the stable climbing stage, where the SP’s levelness is relatively stable and reliable. To mitigate the occurrence of data jumps during multiple stable climbs, we took measures to initialize the monitoring data for each levelness of the SP at the commencement of every stable climb, ensuring the accuracy and consistency of our analysis.

To provide further insight into the study data, we conducted a brief analysis of the monitoring data collected during the stabilized climbing stage. The jacking cylinder stroke values ranged from −551.4 to 551.79, the jacking cylinder pressure values ranged from 0 to 53.4, and the SP levelness values ranged from −39.12 to 40.48.

## 3. Methodology

Selecting an appropriate machine learning algorithm is crucial to extracting valuable insights from sensor-monitoring data during the HBM-climbing process. This algorithm should uncover the hidden data and establish a mapping relationship between the HBM climbing parameters and postures. In order to accurately predict postures in HBMs, the chosen model must effectively handle MTS features and automatically capture the nonlinear relationships present in the data. This study employs three MTS neural networks—LSTM, GRU, and TCN—to predict the postures during HBM climbing.

### 3.1. MTS-Prediction Model

#### 3.1.1. LSTM Model

The LSTM model, introduced by Hochreiter and Schmidhuber (1997) [47], addresses the limitations of traditional RNNs by incorporating explicit memory-management mechanisms. By explicitly adding and subtracting information (see Equation (1)) from the LSTM state, the LSTM model ensures that each state cell remains constant over time. Moreover, gated cells and carefully designed memory cells enable the model to preserve its long-term memory while maintaining the relevance of the most recent state. This prevents information distortion, disappearance, and sensitivity explosion, which could occur in other models like Neural Turing Machines

(1) $$S_{t}=S_{t-1}+\Delta S_{t}$$

**Figure 5:**
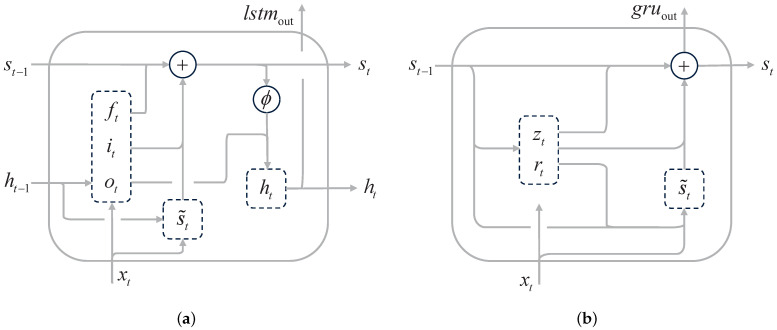
Diagram of MTS prediction RNNs model: (**a**) schematic diagram of LSTM model, (**b**) schematic diagram of GRU model.

A typical LSTM cell, as illustrated in Figure 5a, consists of three key gates: the forget gate, the input gate, and the output gate. These gates play a critical role in managing the memory of the network by modulating the activation of the weighted sum function W(∗). As depicted in Equation (2) [47], the forget gate $f_{t}$ decides which information should be retained or discarded. It utilizes a sigmoid function ($\sigma \left(x\right)=\frac{1}{1+e^{-x}}$) to compute a vector (output) based on the previous cell state $s_{t-1}$ and the current input information $x_{t}$. The purpose of this scaling is to rescale the values of each dimension of the data within the range of [0,1]. Subsequently, the sigmoid function σ(x) determines the relevance of the current information and determines which information will contribute to the computation of the cell state $s_{t}$. The input gate $i_{t}$ determines the information that should be stored in the cell state. It employs the sigmoid function σ(x) to compute its activation based on the previous cell state $s_{t-1}$ and the current input information $x_{t}$. The candidate cell state ${\tilde{s}}_{t}$ is computed using the hyperbolic tangent function ($\phi \left(x\right)=\frac{e^{x}-e^{-x}}{e^{x}+e^{-x}}$), which yields a value ranging from −1 to +1. The outputs of these computations ($s_{t-1}$ and ${\tilde{s}}_{t}$) are combined to update the current internal cell state $s_{t}$. Lastly, the output gate $o_{t}$ determines the final output $lstm_{out}$ and the next hidden state $h_{t}$. The output gate $o_{t}$ multiplies the previous hidden state $h_{t-1}$ and the current input information $x_{t}$ with the sigmoid function σ(x)-activation output. These calculations determine the information that the hidden layer $h_{t}$ will carry.

(2) $$\left\{\begin{array}{l} \begin{array}{cc} f_{t} & =\sigma \left(W_{f}s_{t-1}+U_{f}x_{t}+b_{f}\right)\\ i_{t} & =\sigma \left(W_{i}s_{t-1}+U_{i}x_{t}+b_{i}\right)\\ o_{t} & =\sigma \left(W_{o}s_{t-1}+U_{o}x_{t}+b_{o}\right) \end{array}\\ \begin{array}{c} {\tilde{s}}_{t}=\phi \left(W\left(o_{t}⊙s_{t-1}\right)+Ux_{t}+b\right)\\ s_{t}=f_{t}⊙s_{t-1}+i_{t}⊙{\tilde{s}}_{t}\\ h_{t}=o_{t}\phi \left(s_{t}\right) \end{array} \end{array}\right.$$

#### 3.1.2. GRU Model

The GRU model (Cho et al., 2014 [39]) is a simplified variant of the RNNs architecture. Unlike the LSTM model, the GRU model consists of only two gates: the reset gate and the update gate. Figure 5b illustrates the structure of the GRU model. As shown in Equation (3) [39], in contrast to LSTM, GRU eliminates the need for a separate hidden state *h*. Instead, it directly replaces the input gates $i_{t}$ with $\left(1-z_{t}\right)$, where $z_{t}$ represents the update gate. The reset gate $r_{t}$ determines the combination of the input information $x_{t}$ and the previous cell state $s_{t-1}$. The update gate $z_{t}$ controls the retention of the previous cell state $s_{t-1}$. The cell state $s_{t}$ is then updated by combining all the computed information.

(3) $$\left\{\begin{array}{c} r_{t}=\sigma \left(W_{r}s_{t-1}+U_{r}x_{t}+b_{r}\right)\\ z_{t}=\sigma \left(W_{z}s_{t-1}+U_{z}x_{t}+b_{z}\right)\\ {\tilde{s}}_{t}=\phi \left(W\left(r_{t}⊙s_{t-1}\right)+Ux_{t}+b\right)\\ s_{t}=z_{t}⊙s_{t-1}+\left(1-z_{t}\right)⊙{\tilde{s}}_{t} \end{array}\right.$$

#### 3.1.3. The TCN Model

**Figure 6:**
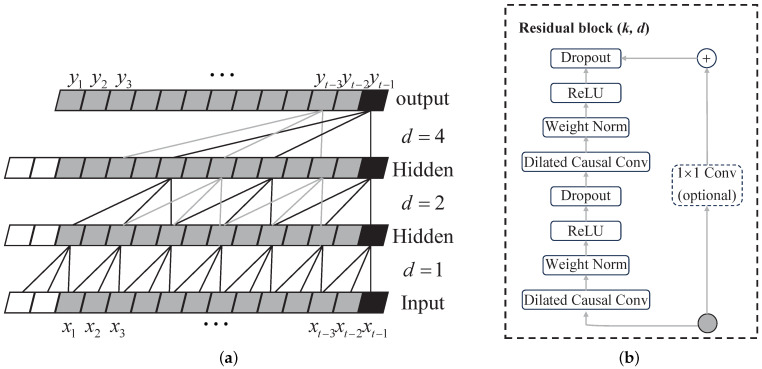
Schematic diagram of TCN model: (**a**) dilated causal convolution, (**b**) residual block network structure.

The TCN model (Bai et al., 2018 [43]) is a one-dimensional dilated causal-convolutions neural network (CNN) designed for solving time series problems. It incorporates the architecture of CNNs and introduces the concept of dilated causal convolutions. Figure 6 illustrates the main components of the TCN model. The TCN model utilizes a one-dimensional dilated causal convolution with a filter size of 3 and a residual network structure. The combination of causal convolution and dilated convolutions allows the convolutional layer to expand its receptive field while strictly adhering to temporal constraints. This larger receptive field enables the model to learn and extract historical information from multivariate time series data. The expansion convolution operation H(S) is employed in the sequence unit *S* to process a one-dimensional time series input $X=(x_{1},x_{2},\cdots ,x_{t-1})$, where f:{0,1,⋯,k−1} is the filter. The operation is defined as [43]

(4) H(S)=(X*fd)(S)=∑i=0k−1f(i)·XS−d·i

where *S* represents the input sequence information, ∗ is the convolution operator, *k* denotes the filter size, $d=2^{v}$ represents the dilation factor, where *i* is the levelness of the network, and S−d·i indicates the localization of specific historical information.

Additionally, the TCN model incorporates a residual network structure, which helps address issues like gradient vanishing or explosion and model degradation during deep neural network training. Typically, the TCN model consists of two layers of residual modules. Each module comprises dilated causal convolution, weight normalization, ReLU-activation function (f(x)=max(0,x), where *x* is the input), and dropout. The previous layer’s output serves as the input to the dilated causal convolution of the next layer, ensuring that both the inputs $x_{i}$ and outputs $x_{i+1}$ of the module have the exact dimensions. An additional 1×1 convolution is applied to restore the original number of channels.

(5) $$x_{i+1}=f\left(X+F\left(X\right)\right)$$

where F(∗) refers to a series of transformations leading from one residual module to another.

### 3.2. The Architecture for the Posture-Prediction Model of HBMs

#### 3.2.1. Data Standardization and Set Partitioning

The data obtained from different sensors often have varying size ranges, resulting in sample data in the dataset having different scales. We perform standardized preprocessing on the raw data to address this issue and prevent model bias towards certain features due to the magnitude differences during training. In this experiment, we apply min–max normalization to the sensor data samples, resulting in a value range of [−1, 1]. After normalization, the scaled values of the sensor data samples $X_{\mathrm{norm}}$ or $Y_{\mathrm{norm}}$ (where *X* represents the active input dataset and *Y* represents the passive output dataset) can be expressed as follows:

(6) $$X_{\mathrm{norm}}=\frac{X-X_{\min}}{X_{\max}-X_{\min}}$$

where $X_{\max}$ denotes the maximum value of the sample dataset, and $X_{\min}$ denotes the minimum value of the sample dataset.

The dataset is partitioned into a training set (80%), a validation set (10%), and a test set (10%). The training set is utilized to train the neural network model. The validation set is used to assess the performance of the untrained model, serving as a means to prevent overfitting and underfitting. Finally, the test set evaluates the optimal model’s accuracy, precision, and recall performance metrics.

#### 3.2.2. The Evaluation of Predictive Performance

In this study, the mean absolute error (MAE) and coefficient of determination for Goodness of Fit (R^2^) is used as an evaluation metric for the prediction model:

(7) $$\begin{array}{c} \mathrm{MAE}=\frac{1}{m}\sum_{i=1}^{m}\left|y_{i}-{\hat{y}}_{i}\right|\\ R^{2}=1-\frac{\sum_{i=1}^{m}{\left(y_{i}-{\hat{y}}_{i}\right)}^{2}}{\sum_{i=1}^{m}{\left(y_{i}-{\bar{y}}_{i}\right)}^{2}} \end{array}$$

where *m* represents the total number of moments, $y_{i}$ denotes the actual value at moment *i*, ${\hat{y}}_{i}$ refers to the predicted value of the model output at moment *i*, and ${\bar{y}}_{i}$ denotes the average of all actual values.

The MAE can better reflect the reality of prediction-value errors. A more petite MAE indicates a better prediction effect. On the other hand, R^2^ ranges between 0 and 1, where a value closer to 1 indicates a better fit of the model to the data, suggesting a more vital predictive capability.

#### 3.2.3. MTS-Prediction Architecture

**Figure 7:**
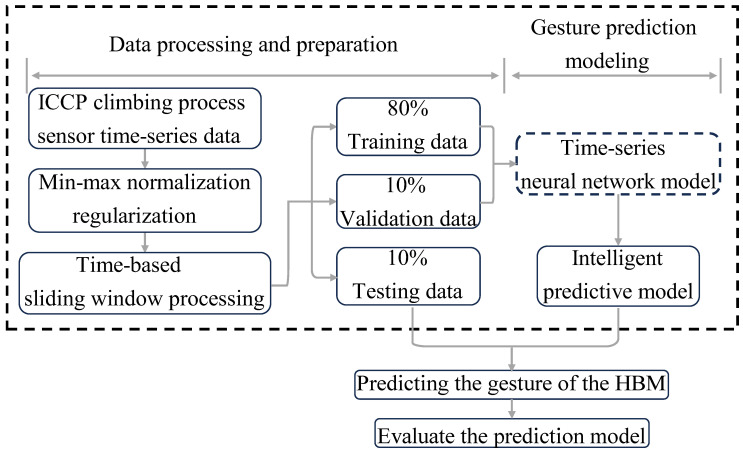
Flowchart of the posture-prediction model framework for the HBM-climbing process.

The flowchart in Figure 7 illustrates the technical route employed in this study to predict the HBM postures. Initially, the sensor-monitoring data of the HBM is preprocessed. Subsequently, a MTS neural network model is trained using the training set data, while the validation-set data is utilized for model selection and fine-tuning. This experiment employs the root mean square error (RMSE) as the loss function:

(8) $$\mathrm{RMSE}=\sqrt{\frac{1}{m}\sum_{i=1}^{m}{\left(y_{i}-{\hat{y}}_{i}\right)}^{2}}$$

In addition, the Adam optimizer was used to update the network parameters for model training and model output.

**Figure 8:**
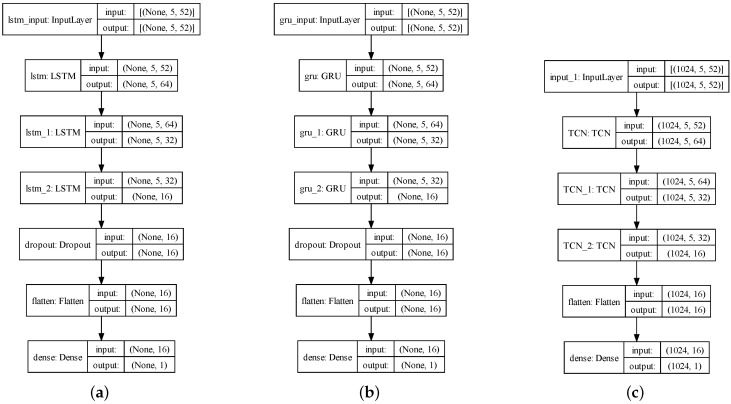
MTS neural network-model architecture: (**a**) LSTM model architecture, (**b**) GRU model architecture, (**c**) TCN model architecture.

Figure 8 illustrates this study’s three MTS neural network-model architectures. Notably, there are similarities between the LSTM and GRU model architectures. The LSTM, GRU, and TCN functions are utilized in the hidden layers of these neural network-model architectures. The weight initialization techniques used for all three functions are the commonly used Xavier initialization, including the initialization of input gate, forget gate, output gate, and candidate memory cell weights in the LSTM function, the initialization of reset gate, update gate, and candidate hidden state weights in the GRU function, and the initialization of one-dimensional convolution kernel, residual block, and fully connected layer weights in the TCN function. All three models in this experiment use 3 hidden layers to make the prediction results are comparable and set the same learning rate η=0.01. In order to prevent overfitting, the Dropout function (1−p=0.2) is employed as a regularization technique. It is worth mentioning that both the LSTM and GRU models implement the Dropout function after the hidden layers, while the TCN model incorporates the Dropout function within each TCN function. Finally, a fully connected layer is employed to achieve a dimensionality reduction in the output.

## 4. Results

### 4.1. Sensitivity Analysis

This study selected 26 jacking cylinder strokes and 26 jacking cylinder pressures as the active inputs *X* for the multivariate analysis of HBM-climb posture-prediction. The levelness of 16 monitoring points on the SP during the HBM climb was chosen as the passive output *Y*.

**Figure 9:**
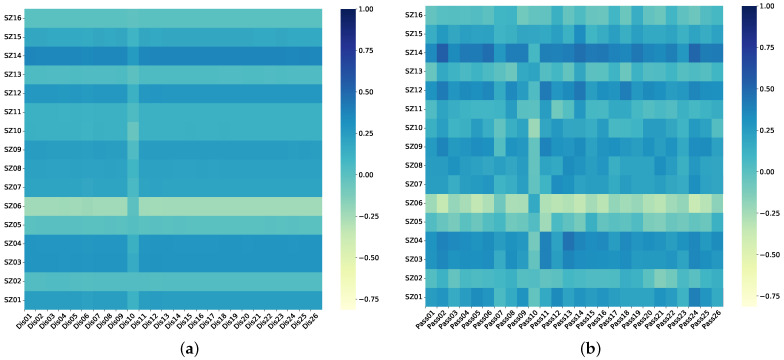
Sensitivity analysis of sensor-monitoring information during climbing of HBM: (**a**) Sensitivity between jacking cylinder stroke and SP levelness, (**b**) sensitivity between jacking cylinder pressures and SP levelness.

Figure 9a,b illustrates the Pearson correlation coefficients $\rho_{X,Y}$ (see Equation (9)) between the jacking cylinder stroke and the SP levelness, and between the jacking cylinder pressure and the SP levelness, separately. The Pearson correlation coefficient provides insight into the relationship between the jacking cylinder stroke, jacking cylinder pressure, and the levelness of each position on the SP. Notably, the displacements of the SP at SZ01, SZ03, SZ04, SZ07, SZ08, SZ09, SZ12, and SZ14 show a more pronounced response to the activity of the jacking cylinder.

(9) $$\rho_{X,Y}=\mathrm{corr}\left(X,Y\right)=\frac{\mathrm{cov}(X,Y)}{\sigma_{X}\sigma_{Y}}=\frac{E\left[\left(X-\mu_{X}\right)\left(Y-\mu_{Y}\right)\right]}{\sigma_{X}\sigma_{Y}}$$

where cov(∗,∗) is the covariance and σ is the standard deviation.

### 4.2. Model Comparison and Evaluation

In this study, a posture-prediction model for HBM is constructed using the LSTM neural network, GRU neural network, and TCN neural network. These models were trained and validated using separate datasets. After 200 epochs with a batch size of 1024, the models generated an intelligent prediction model for the 16-point levelness of the SP in HBM.

**Figure 10:**
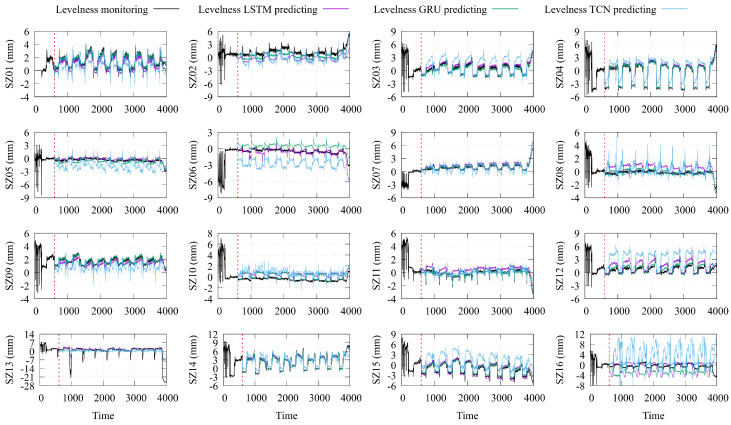
Predicted SP levelness using LSTM, GRU, and TCN models.

The intelligent prediction model takes historical time series values of the monitored stroke and pressure of the jacking cylinder as input, and the predicted time series values of steel platform levelness as output. A test dataset was used to validate the final HBM posture-prediction model. The prediction results of the LSTM, GRU, and TCN models were compared with the actual levelness of the 16 monitoring points on the construction platform, as shown in Figure 10.

Based on the fluctuating patterns and peaks of the overall levelness, the LSTM and GRU models exhibited a certain levelness of reliability in predicting the HBM postures. However, the TCN model demonstrated relatively more significant prediction errors. An in-depth analysis was conducted to evaluate the accuracy and predictive ability of the three intelligent-prediction models. The goodness-of-fit correlation coefficient (R^2^) and the mean absolute error (MAE) (see Equation (7)) were used as performance indicators, as detailed in Table 1. The TCN model consistently exhibited MAE values generally exceeding 1.0, indicating a substantial deviation between the predicted values and the actual monitoring values. Similarly, the R^2^ values for the TCN model were predominantly below 0.6, suggesting a lack of compatibility. For the LSTM model, MAE values surpassing 1.0 were observed at monitoring points SZ13, SZ15, and SZ16, with a significant error of 7.305 at SZ13. The corresponding R^2^ values were less than 0.8 at SZ06, SZ10, SZ13, and SZ16, indicating poor fitting at these monitor points. The GRU model only exhibited a MAE value greater than 1.0 at monitoring point SZ13, reaching 1.128. Moreover, the R^2^ values at SZ05, SZ06, and SZ16 fell below 0.8, indicating a weaker fit at these monitoring points. Notably, the R^2^ value at SZ16 was a mere 0.268, suggesting an almost complete lack of fitting ability at this point.

Observing the prediction results of the LSTM and GRU models, although not all the predicted values exactly matched the real levelness, the levelness of the building-steel platforms at points SZ01, SZ03, SZ04, SZ07, SZ08, SZ09, SZ12, SZ14, and SZ15 showed a better prediction effect, with similar fluctuation patterns and peaks in the predicted and actual monitoring values. This further validates the results of the sensitivity analysis between multiple explanatory variables and multiple observed variables in Section 4.1.

**Figure 11:**
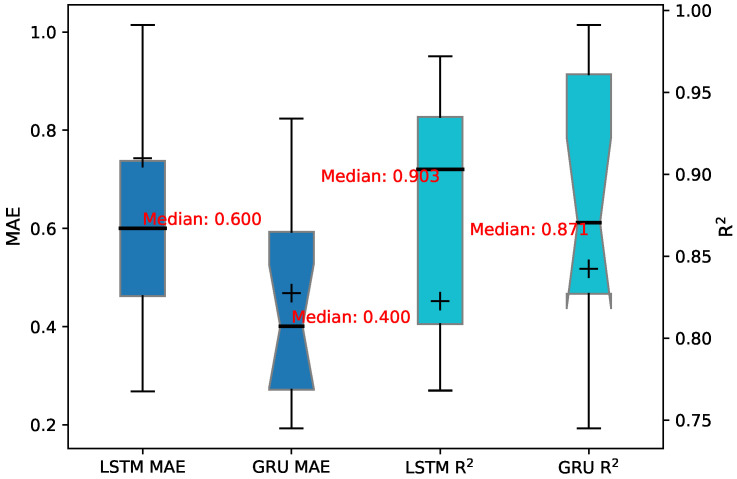
MAE and R^2^ under LSTM and GRU model prediction.

Figure 11 also demonstrates that the GRU model consistently had smaller MAE values, indicating higher accuracy in predicting the HBM posture. On the other hand, the LSTM model showcased R^2^ values closer to 1 across all instances, indicating a better fit to the HBM posture.

However, monitoring points SZ10, SZ13, and SZ16 exhibited poor predictive performance across all three intelligent-prediction models, indicating a lack of sensitivity to jacking cylinder data and susceptibility to other influencing factors. The unsatisfactory prediction outcomes of the TCN model imply an inability to capture the significance of long-term features, requiring further enhancements such as introducing larger convolution kernels to capture the long-term trends of HBM postures.

## 5. Discussion

In this research, we focused on utilizing various neural network models, including LSTM, GRU, and TCN, to predict changes in the postures of the HBM based on historical data on the levelness of SPs, stroke, and the pressure of climbing jacking cylinders. We employed a multivariate time series-related model approach to address the challenge of multiple output prediction. We conducted a thorough analysis by comparing and evaluating the prediction outcomes of these time series neural network methods. We selected the optimal solution based on calculating the mean square error, which effectively reduced model randomness and improved the correlation of levelness at each monitoring point of the HBM. By training the multivariate time series neural network, we successfully obtained a model capable of predicting the postures of the HBM. Leveraging the historical monitored data during the climbing process, our model enabled accurate predictions of subsequent data. Thus, the necessary adjustments were made to each of the jacking cylinders, even when the load distribution of the SP remained unknown.

**Figure 12:**
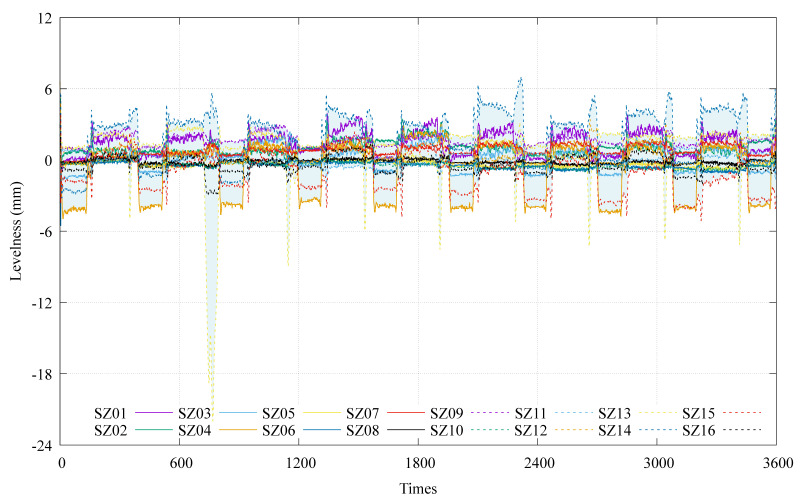
Use of hydrostatic levelness sensors to monitor the time course of the levelness curves at different positions of the steel platform during the climbing process.

During the climbing of the HBM, it is imperative to minimize the variation in the SP’s postures. Drawing upon the comprehensive analysis presented earlier, it becomes clear that the GRU model exhibits superior accuracy in predicting the hand postures of the HBM. Consequently, the HBM operator can leverage the intelligent-prediction model with the GRU neural network to anticipate future changes in HBM postures during the climbing process. Moreover, it enables a continuous adjustment of the HBM’s postures in response to the predicted outcomes. Figure 12 illustrates the temporal data obtained from 16 levelness sensors during the climbing of the HBM. As the HBM climbs, the readings from the 16 sensors consistently demonstrate changes in the measured levelness. Furthermore, the levelness deviations across the 16 positions on the SP undergo dynamic changes in conjunction with the climbing process.

During the intricate process of climbing the HBM, the stroke of the jacking cylinder directly controls the HBM’s posture while significantly impacting its overall posture dynamics. So, it is clearly suboptimal to rely on adjusting the stroke of the jacking cylinder to rectify the HBM’s posture. In order to further leverage the proposed model to offer guidance on the HBM’s climbing state, we engage in a dynamic adjustment of the jacking pressure values across the jacking cylinders of the various positions. This approach enables us to dynamically rectify the HBM’s postures, thereby minimizing the levelness deviations among different positions on the SP. Figure 9b showcases the results of a Pearson correlation analysis conducted between the levelness of the SP and the pressure exerted by the jacking cylinder. Remarkably, there is always the highest correlation discernible between the fluctuations observed in the levelness at each position on the SP and the pressure values of one jacking cylinder. Consequently, when encountering instances where the levelness at a particular position is suboptimal, we adjust the corresponding jacking cylinder’s pressure value, enhancing the levelness, diminishing the deviation in levelness across the SP, and rectifying the HBM’s posture.

**Figure 13:**
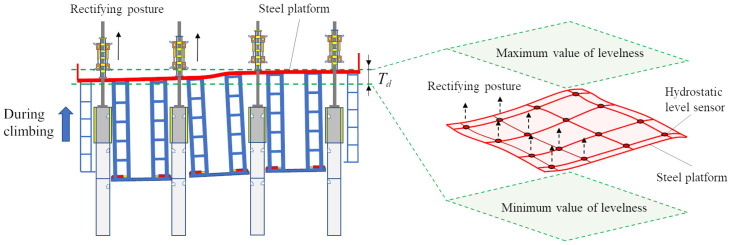
Schematic diagram of the posture adjustment during HBM climbing process.

In summary, in situations where significant deviations in the HBM’s postures manifest, we can minimize the levelness deviations on the SP by augmenting the pressure value of the jacking cylinder, which has a positive correlation with the low-levelness position point on the SP. This approach allows us to rectify the HBM’s postures and attenuate the levelness deviations. By harnessing the predictive capabilities of the proposed model, we can anticipate the forthcoming changes in the HBM’s posture. This integration of the model into the HBM’s intelligent system empowers the HBM to adjust its climbing parameters autonomously, ensuring the smooth and secure operation of the HBM. Figure 13 visually illustrates the process of adjusting the hand postures of the HBM during its climbing. Throughout the actual climbing process, it is imperative to minimize the levelness deviations among each monitoring position on the SP. Furthermore, it is crucial to maintain the maximum and minimum values of levelness deviations within a threshold $\left(T_{d}\right)$ to mitigate the risk of the tube structure colliding with the reinforced concrete main structure of the building. Such collisions can lead to instability in the climbing process, posing a potential hazard to the HBM. The proposed model not only accurately predicts the climbing postures of the HBM but also provides specific adjustment recommendations to ensure the safe operation of the HBM and mitigate construction risks.

## 6. Conclusions

In this paper, we propose a multivariate time series neural network prediction system to control the HBM and maintain a smooth posture during construction operations. The system utilizes three multivariate time series neural network models: LSTM, GRU, and TCN. The pressure and stroke of the jacking cylinder serve as inputs, while the monitored values of sensor levelness at 16 different positions on the SP are used as outputs. The main objective is to address the issue of unstable posture in the HBM caused by significant levelness deviations resulting from uneven stacking on the SP during the climbing process. By predicting future posture changes based on the climb data from the historical working stage of the HBM, we aim to proactively control the levelness deviations of the SP within a threshold value and prevent instability in the HBM’s posture during the climbing process. The results of the study are as follows:

1. For the same neural network architecture and dataset size, the prediction system that uses the GRU neural network model does a better job of guessing how the HBM posture will change while it climbs. Among the multiple levelness sensors installed on the HBM, only a subset of them demonstrate a strong correlation with the jacking parameters of the jacking mechanism. By adjusting the pressure value of the jacking cylinders, the posture of the HBM can be conveniently corrected. Therefore, we propose employing the GRU neural network-prediction system to anticipate the posture changes of the HBM. Additionally, by adjusting the jacking cylinder pressure value, it is possible to maintain levelness within the threshold value;
2. The validation of the measured data demonstrates that the proposed prediction models can accurately determine the levelness deviations and posture of the HBM’s steel platform by solely utilizing the working data from the jacking cylinders. This capability allows for real-time warnings, indicating that these networks can make significant contributions to the safe and efficient operation of the HBM. Moreover, this modified method can also be extended to monitor the operational status of other engineering equipment, such as hydraulic climbing molds, sliding molds, and integral lifting scaffolds. The widespread adoption and implementation of this method could improve the construction levelness of high-rise buildings;
3. However, it is important to note that the model was trained and tested solely based on data from a single HBM, and its applicability to other HBMs or construction platforms has yet to be verified. Additionally, the model primarily considers the pressure and stroke of the jacking cylinder as inputs without accounting for the potential influence of other environmental factors, such as weather conditions. Finally, while the GRU provided the best prediction in this study, it should not be assumed that the GRU is the optimal choice in all scenarios. Therefore, future studies should improve the preprocessing and cleaning of the data and validate the generalization ability of these models. More characteristic factors should be considered under a wider range of equipment and conditions for different types of HBM operational data. This approach will lead to more comprehensive and high-performance predictive models;
4. The developed models offer real-time predictions to site managers and operators, allowing them to understand the HBM’s status during the climbing process promptly. This timely understanding enables them to make the necessary adjustments in accordance with HBM management requirements and standard specification terms, thus ensuring a safer and more efficient climbing process.

## Figures and Tables

**Table 1:** MAE and R^2^ of prediction using LSTM, GRU, and TCN models.

	LSTM MAE	LSTM R^2^	GRU MAE	GRU R^2^	TCN MAE	TCN R^2^
SZ01	0.599	0.927	0.274	0.870	1.784	0.263
SZ02	0.484	0.813	0.345	0.860	1.536	0.034
SZ03	0.601	0.944	0.593	0.961	1.294	0.612
SZ04	0.581	0.963	0.593	0.961	1.510	0.731
SZ05	0.795	0.821	0.456	0.745	1.069	0.267
SZ06	0.522	0.795	0.625	0.585	1.995	−0.934
SZ07	0.395	0.932	0.593	0.961	1.445	0.065
SZ08	0.364	0.903	0.224	0.815	0.680	0.671
SZ09	0.268	0.972	0.193	0.854	1.269	0.296
SZ10	0.718	0.768	0.304	0.871	1.266	0.243
SZ11	0.316	0.903	0.261	0.959	0.855	0.641
SZ12	0.626	0.970	0.298	0.831	3.435	0.117
SZ13	7.305	0.462	1.128	0.988	9.923	0.071
SZ14	0.656	0.916	0.515	0.957	2.072	0.350
SZ15	1.014	0.871	0.264	0.991	2.026	0.613
SZ16	1.975	−0.207	0.824	0.268	6.316	−8.734

## Data Availability

The data presented in this study are available upon request from the corresponding author. The data are private since the data would reveal the operation of the integral climbing construction platform.

## Abbreviations

The following abbreviations are used in this manuscript:

HBM	High-rise building machine
SP	Steel platform
NNs	Neural networks
RNN	Recurrent neural network
LSTM	Long short-term memory
GRU	Gated recurrent unit
TCN	Temporal convolutional neural network
MTS	Multivariate time series
MAE	Mean absolute error
RMSE	Root mean square error
R^2^	R-squared coefficient
